# New Hippocampal Neurons Mature Rapidly in Response to Ketamine But Are Not Required for Its Acute Antidepressant Effects on Neophagia in Rats^1^,^2^,^3^

**DOI:** 10.1523/ENEURO.0116-15.2016

**Published:** 2016-03-31

**Authors:** Amelie Soumier, Rayna M. Carter, Timothy J. Schoenfeld, Heather A. Cameron

**Affiliations:** Section on Neuroplasticity, National Institute of Mental Health, National Institutes of Health, Bethesda, Maryland 20892

**Keywords:** antidepressive agents, cell proliferation, dentate gyrus, mood disorders, neurogenesis, neuronal maturation

## Abstract

Virtually all antidepressant agents increase the birth of granule neurons in the adult dentate gyrus in rodents, providing a key basis for the neurogenesis hypothesis of antidepressant action. The novel antidepressant ketamine, however, shows antidepressant activity in humans within hours, far too rapid for a mechanism involving neuronal birth. Ketamine could potentially act more rapidly by enhancing maturation of new neurons born weeks earlier. To test this possibility, we assessed the effects of S-ketamine (S-(+)-ketamine hydrochloride) injection on maturation, as well as birth and survival, of new dentate gyrus granule neurons in rats, using the immediate-early gene zif268, proliferating cell nuclear antigen, and BrdU, respectively. We show that S-ketamine has rapid effects on new neurons, increasing the proportion of functionally mature young granule neurons within 2 h. A single injection of S-ketamine also increased cell proliferation and functional maturation, and decreased depressive-like behavior, for at least 4 weeks in rats treated with long-term corticosterone administration (a depression model) and controls. However, the behavioral effects of S-ketamine on neophagia were unaffected by elimination of adult neurogenesis. Together, these results indicate that ketamine has surprisingly rapid and long-lasting effects on the recruitment of young neurons into hippocampal networks, but that ketamine has antidepressant-like effects that are independent of adult neurogenesis.

## Significance Statement

Ketamine is a novel antidepressant agent that works very rapidly. Although ketamine acts as an antagonist of NMDA-type glutamate receptors, it is not clear how or where in the brain it acts to produce its antidepressant effects. This study demonstrates that ketamine has very rapid effects on the maturation of hippocampal neurons born in the adult brain, which have been linked to depression. However, behavioral experiments showed antidepressant-like effects of ketamine on neophagia that are independent of new neurons, in contrast to the effects of classic antidepressants on this behavior. Ketamine effects on new neurons should be considered as potential side effects of treatment and also point to a role for NMDA receptors in the normal maturation of new neurons.

## Introduction

Major depression is a complex and disabling psychiatric disorder that is commonly treated with monoaminergic agents, such as serotonin-selective reuptake inhibitors (SSRIs) or norepinephrine-selective reuptake inhibitors. These standard antidepressant agents have, in addition to limited therapeutic efficacy, delayed onset of action, requiring several weeks of treatment to produce clinical improvement (Browne and Lucki, 2013; Abdallah et al., 2015; Iadarola et al., 2015). These limitations point to the need for more effective and faster-acting antidepressant treatments.

Beyond the monoaminergic system, accumulating evidence supports a role for glutamatergic transmission in depression. Abnormalities in glutamate levels in plasma and brain tissue, as well as alteration in glutamate (AMPA, kainate, NMDA) receptor function have been reported in depressed patients (Sanacora et al., 2008). Evidence suggests that adaptive changes in NMDA receptor expression and function may even represent a final common pathway for monoaminergic antidepressants (Skolnick et al., 1996, 2009). Supporting the idea that NMDA receptors represent promising antidepressant targets, several clinical studies have reported an antidepressant response to a single low-dose infusion of ketamine, a noncompetitive NMDA receptor antagonist (Abdallah et al., 2015; Iadarola et al., 2015). Remarkably, antidepressant activity is observed in depressed patients resistant to prior treatments, begins in <4 h, and is relatively sustained, lasting at least 1-2 weeks (Abdallah et al., 2015; Iadarola et al., 2015). Antidepressant effects of ketamine at subanesthetic doses have also been reported (Browne and Lucki, 2013) in rodent models of antidepressant efficacy, such as the forced swim and tail suspension tests. Preclinical studies (Browne and Lucki, 2013; Duman, 2014) suggest that ketamine may exert its antidepressant activity through alterations in AMPA receptors, BDNF, mammalian target of rapamycin, and glycogen synthase kinase-3, and the formation of new dendritic spines and synapses in the prefrontal cortex. However, other evidence points to a role for the hippocampus in the antidepressant effects of ketamine (Kavalali and Monteggia, 2015).

A key feature of the hippocampus is the ongoing production of granule neurons in the dentate gyrus throughout life. The now well known “neurogenesis hypothesis” of antidepressant action has linked adult neurogenesis to mood disorders and their treatment (Duman et al., 2001; Warner-Schmidt and Duman, 2006). Six classes of monoaminergic antidepressants all increase the proliferation of granule cell precursors (Duman, 2004). Importantly, the delayed onset of antidepressant action parallels the time course of changes in neurogenesis. Although SSRIs immediately change extracellular levels of serotonin, several weeks of SSRI treatment are required to improve clinical symptoms in humans or to increase neurogenesis in animals (Malberg et al., 2000; Warner-Schmidt and Duman, 2006). Even if antidepressants were to rapidly increase cell proliferation, the additional new neurons would likely not contribute to behavior for several weeks after maturation and functional integration into the local hippocampal network (Piatti et al., 2006; Snyder et al., 2009b). This maturation delay suggests that the rapid behavioral effects of ketamine treatment are not due to the birth of new neurons.

Acceleration of neuronal maturation, a much later stage in the neurogenic process, may represent an important target for novel rapidly acting antidepressants. Previous studies have found that some antidepressants accelerate maturation after long-term treatment. Agomelatine, a novel antidepressant with mixed MT1/MT2 melatonin receptor agonist and 5-HT_2C_ receptor antagonist properties, accelerates the maturation of young granule cells after 8 d of treatment (Soumier et al., 2009). Long-term treatment with the SSRI fluoxetine also facilitates the functional maturation of newly generated immature neurons after 21 d, but not after 5 d, of treatment, paralleling its effects on behavior (Wang et al., 2008). One of the fastest and most effective antidepressant treatments is electroconvulsive therapy (Warner-Schmidt and Duman, 2006); electroconvulsive seizures also stimulate dendritic outgrowth and maturation (Overstreet-Wadiche et al., 2006). Together, these studies suggest that the acceleration of hippocampal granule cell maturation could produce antidepressant effects. Therefore, we hypothesized that ketamine might rapidly enhance neuronal maturation to immediately increase the pool of functional young neurons in the hippocampus and that this increase could reduce depressive-like behaviors.

We assessed the rapid and sustained effects of S-ketamine (S-(+)-ketamine hydrochloride, Sigma-Aldrich), an enantiomer of ketamine with robust effects and possibly fewer side effects (Mathew et al., 2012), in rodent behavioral tests that normally require long-term antidepressant treatment. We then determined the short-term effects of S-ketamine on the functional maturation of young neurons and the long-term effects of S-ketamine on granule cell birth, maturation, and survival. Finally, we tested the role of new neurons in the antidepressant-like action of ketamine on novelty-suppressed feeding (NSF).

## Materials and Methods

### Animals

For most experiments, adult (8-week-old) male Long–Evans rats were ordered from a vendor (Charles River Laboratories) and pair housed under a standard 12 h light/dark cycle with free access to food and water for at least 1 week prior to the start of experiments. For testing the role of neurogenesis in NSF behavior, rats expressing herpes simples virus–thymidine kinase (HSV-TK rats) under the GFAP promoter (GFAP-TK rats) were generated on a Long–Evans background, using a construct previously used to make transgenic mice (Snyder et al., 2011). Male offspring were genotyped by PCR after weaning, and wild-type and transgenic littermates were randomly pair housed under a standard 12 h light/dark cycle for the duration of the experiment. All animal procedures were performed in accordance with the Institute of Laboratory Animal Research guidelines and were approved by the Animal Care and Use Committee of the National Institute of Mental Health.

### Drug treatments

#### Experiment 1: behavioral effects of S-ketamine

##### Experiment 1a: novelty-suppressed feeding

Rats were deprived of food for 24 h. S-ketamine at one of three doses (2.5, 5, or 10 mg/kg, all 2 ml/kg in 0.9% saline, i.p.) or saline was injected 1 h prior to testing. The highest dose used here, 10 mg/kg, is 7.5-10× lower than anesthetic doses in rats; racemic mixtures at this dose produce antidepressant-like effects without altering spontaneous locomotor activity (Hunt et al., 2006; Wilson et al., 2007; Garcia et al., 2008; Engin et al., 2009; Li et al., 2010). One hour later, animals were placed in a brightly illuminated opaque Plexiglas box (50 × 50 × 40 cm) with six pellets of regular chow in the center. Behavior was video recorded from above for 10 min, and latency to feed was determined from the recordings.

##### Experiment 1b: forced swim test

Rats were individually placed in a 50-cm-high clear cylinder containing water (23 ± 1°C, 30 cm depth) for 15 min. One day later, rats were placed in the water again for a 5 min test. Each rat received three injections of S-ketamine (10 mg/kg, i.p.; Sigma-Aldrich), fluoxetine (10 mg/kg, i.p.; Sigma-Aldrich), or saline, 24 h, 4 h, and 30 min prior to the test session (Porsolt et al., 2001). To assess the long-term effects, rats were placed in water again for 5 min at 21 d after drug injection. Swim sessions were video recorded from the side, and immobility, swimming, and climbing behaviors were scored at the end of each 5 s period from the recordings (Cryan et al., 2005).

#### Experiment 2: short-term effects of S-ketamine

##### Experiment 2a: effects 16 h after S-ketamine

Rats were injected with bromodeoxyuridine (BrdU; 200 mg/kg, i.p.; 10 mg/ml in saline with 0.007N NaOH) to identify young granule cells for maturation and survival analyses. Beginning 2 d after BrdU injection (Day 2), rats were injected daily with saline for 14 d to match the daily injections used for long-term treatment in Experiment 3. On Day 16, rats were given a single injection of either S-ketamine (10 mg/kg, i.p.; Sigma-Aldrich) or saline. Sixteen hours later (Day 17), they were injected with kainic acid (15 mg/kg, i.p.; Tocris Bioscience) to drive immediate-early gene (IEG) expression in synaptically integrated granule cells (Snyder et al., 2009a,b). Sodium pentobarbital (50 mg/kg, i.p.; Ovation Pharmaceuticals) was given to stop seizures 30 min after the onset of stage 5 seizure activity. Rats were perfused 90 min after the onset of stage 5 seizure onset.

##### Experiment 2b: effects of S-ketamine in 7-d-old cells

Rats were treated exactly as above in Experiment 2a, except that ketamine was injected on Day 7 after BrdU injection, and no saline injections were given on the intervening days.

##### Experiment 2c: effects 2 h after S-ketamine

Rats were treated exactly as above in Experiment 2a, except that ketamine was injected on Day 14 after BrdU injection, no saline injections were given on the intervening days, and kainic acid was injected 2 h after saline or ketamine (also on Day 14).

#### Experiment 3: effects of long-term S-ketamine treatment

Rats were injected with BrdU as described above. Beginning on Day 2 after BrdU injection, rats were injected daily with S-ketamine (10 mg/ml, i.p.; Sigma-Aldrich) or saline. After 14 or 21 d of daily ketamine injection, rats were injected with kainic acid followed by sodium pentobarbital, as described above, and were perfused 90 min after the onset of stage 5 seizure onset.

#### Experiment 4: sustained effects of ketamine in a depression model

##### Experiment 4a: sucrose preference test and survival effects

Rats were injected with BrdU as described above. On Day 2 after BrdU injection, rats were injected once with either S-ketamine (10 mg/kg, i.p.; Sigma-Aldrich) or saline and then were given corticosterone (CORT) in their drinking water (400 µg/ml in 2.5% ethanol/water, v/v, equivalent to 40 mg/kg/d; Sigma-Aldrich) to produce a depression-like state (Sterner and Kalynchuk, 2010). Control rats drank 2.5% ethanol/water. On Day 30, corticosterone and ethanol were removed from the drinking water, and rats were given a sucrose solution (1% in drinking water; Sigma-Aldrich) in one bottle in addition to normal drinking water for a 48 h habituation period. The location of the sucrose bottles (left/right) was balanced across animals and was alternated after 24 h. After 4 h of water deprivation at the beginning of dark phase, rats were given a 1 h free choice test with two identical bottles, one filled with the sucrose solution and the other with water. Sucrose preference was calculated as the volume of sucrose solution over total fluid volume consumed. Rats were perfused on Day 32, just after sucrose preference testing.

##### Experiment 4b: proliferation and maturation effects

Rats were treated as in Experiment 4a above, except that BrdU was injected 16 d after corticosterone was added to the drinking water; there was no behavior testing; and, after 32 d of corticosterone treatment, rats were injected with kainic acid followed by sodium pentobarbital, as described above, and were perfused 90 min after the onset of stage 5 seizure onset.

#### Experiment 5: effects of S-ketamine on behavior in the absence of new neurons

Beginning at 8 weeks of age, GFAP-TK rats and wild-type littermates were given valganciclovir to eliminate adult neurogenesis (100 mg/kg/week, p.o., for 2 weeks, then 20 mg/kg/week for 6 weeks). After 8 weeks of valganciclovir treatment, rats were tested for novelty-suppressed feeding behavior, as described above. Rats were deprived of food for 24 h prior to testing. One hour prior to testing, rats were injected with saline or S-ketamine (10 mg/kg, i.p.; a gift from Irv Wainer, National Institute on Aging, Bethesda, MD). For testing, animals were placed in a brightly illuminated opaque Plexiglas box (50 × 50 × 40 cm) with six pellets of regular chow in the center. Behavior was video recorded from above for 10 min, and the latency to feed was determined from recordings.

### Histological procedures and analysis

Rats were perfused with 4% paraformaldehyde, and brains were sectioned at 40 µm throughout the entire hippocampus. Complete series of sections were enzymatically immunostained for BrdU or proliferating cell nuclear antigen (PCNA), or were fluorescently immunostained for doublecortin (DCX) or BrdU and zif268 combined, using Alexa Fluor dye-conjugated secondary antibodies. zif268 is a synaptic activity-dependent immediate-early gene that is a reliable marker of the maturity of adult-born neurons (Jones et al., 2001; Snyder et al., 2009a,b; Jungenitz et al., 2014). BrdU-labeled (+), PCNA^+^, and DCX^+^ cells in the granule cell layer and subgranular zone were counted stereologically, using a 40× objective, on 1:12 series of sections through the entire dentate gyrus. Cell counts were multiplied by 12 to estimate the total number in each rat. To quantify IEG expression, 25 BrdU^+^ cells per hemisphere from the dorsal dentate gyrus were analyzed for colabeling of zif268 and NeuN^+^ using a 63× objective on a confocal microscope. Statistical analyses were performed using two-way ANOVA with Bonferroni *post hoc* tests, one-way ANOVA followed by the Dunnett’s *post hoc* test, or Student’s *t* test as appropriate (Table 1).

**Table 1:** Statistical table

	Figure	Description	Data structure	Type of test	Power
a	1*A*	Latency to eat in NSF	Normal distribution	ANOVA	*p =* 0.0052
b	1*A*	Latency to eat in NSF (0 vs 10 mg/kg)	Normal distribution	Holm–Sidak *post hoc* test	*p =* 0.0042
c	1*B*	Immobility, main effect of treatment	Normal distribution	2-way repeated measures ANOVA	*p* < 0.0001
d	1*B*	Immobility, main effect of time	Normal distribution	2-way repeated measures ANOVA	*p* = 0.0003
e	1*B*	Immobility, treatment × time interaction	Normal distribution	2-way repeated measures ANOVA	*p =* 0.9980
f	1*B*	Immobility, ketamine vs saline	Normal distribution	Holm–Sidak *post hoc* test	*p* < 0.0001
g	3*A*	Short-term effects on cell maturation	Normal distribution	*t* test	*p* = 0.0375
h	3*B*	Proliferation effects	Normal distribution	*t* test	*p* = 0.0359
i	3*D*	Maturation effects in younger cells	Normal distribution	*t* test	*p* = 0.3548
j	3*F*	Very rapid maturation effects	Normal distribution	*t* test	*p =* 0.0450
k	3*G*	Strong NeuN expression	Normal distribution	*t* test	*p =* 0.0062
l	4*C*	Long-term 14 d maturation effects	Normal distribution	*t* test	*p* = 0.0108
m	4*D*	Long-term 21 d maturation effects	Normal distribution	*t* test	*p* = 0.0181
n	4*E*	Long-term 14 d proliferation effects	Normal distribution	*t* test	*p* = 0.9726
o	4*F*	Long-term 21 d proliferation effects	Normal distribution	*t* test	*p =* 0.365
p	4*G*	Long-term 14 d survival effects	Normal distribution	*t* test	*p* = 0.3280
q	4*H*	Long-term 21 d survival effects	Normal distribution	*t* test	*p* = 0.0191
r	5*A*	Sustained maturation, main effect of CORT	Normal distribution	2-way ANOVA	*p* = 0.9964
s	5*A*	Sustained maturation, main effect of ketamine	Normal distribution	2-way ANOVA	*p* = 0.0017
t	5*A*	Sustained maturation, CORT × ketamine interaction	Normal distribution	2-way ANOVA	*p* = 0.9821
u	5*B*	Proliferation, main effect of ketamine	Normal distribution	2-way ANOVA	*p* = 0.0130
v	5*B*	Proliferation, main effect of CORT	Normal distribution	2-way ANOVA	*p* = 0.0091
w	5*B*	Proliferation, CORT × ketamine interaction	Normal distribution	2-way ANOVA	*p =* 0.9883
x	5*D*	Sucrose preference	Normal distribution	ANOVA	*p* = 0.0072
y	5*D*	Sucrose preference, vehicle/saline vs CORT/saline	Normal distribution	Holm-Sidak *post hoc* test	*p =* 0.0074
z	5*D*	Sucrose preference, CORT/saline vs CORT/ketamine	Normal distribution	Holm-Sidak *post hoc* test	*p =* 0.0405
aa	5*E*	Survival	Normal distribution	ANOVA	*p* = 0.3512
bb	6*D*	DCX+ cell number, main effect of genotype	Normal distribution	2-way ANOVA	*p* < 0.0001
cc	6*D*	DCX+ cell number, main effect of ketamine	Normal distribution	2-way ANOVA	*p* = 0.2393
dd	6*D*	DCX+ cell number, genotype × ketamine interaction	Normal distribution	2-way ANOVA	*p* = 0.2477
ee	6*E*	Latency to eat in NSF, main effect of genotype	Normal distribution	2-way ANOVA	*p* = 0.6008
ff	6*E*	Latency to eat in NSF, main effect of ketamine	Normal distribution	2-way ANOVA	*p* = 0.0016
ee	6*E*	Latency to eat in NSF, genotype × ketamine interaction	Normal distribution	2-way ANOVA	*p* = 0.5469

## Results

### Rapid and prolonged effects of ketamine on behavior

**Figure 1.**
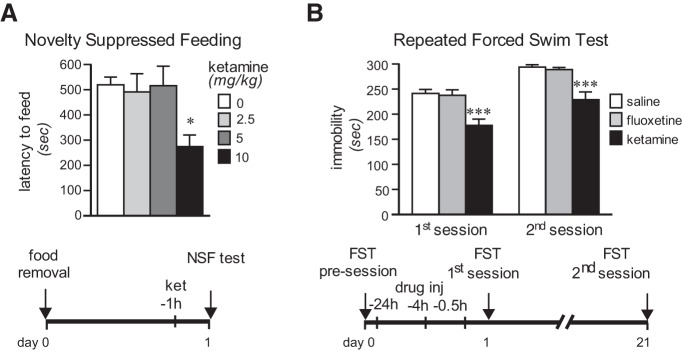
Rapid and sustained behavioral effects of S-ketamine. ***A***, Short-term S-ketamine (ket) treatment reduced the latency to eat in the novelty-suppressed feeding test (one-way ANOVA, *F*_(3,14)_ = 6.61, *p* = 0.005; *Holm–Sidak test, 10 mg/ml vs saline, *p* = 0.004). ***B***, In the repeated forced swim test, short-term administration of S-ketamine reduced the time spent immobile immediately and 21 d later, while fluoxetine had no effect (two-way repeated-measures ANOVA; treatment effect: *F*_(2,9)_ = 31.65, *p* = 0.0001; time effect: *F*_(1,9)_ = 31.47, *p* = 0.0003; treatment × time interaction: *F*_(2,9)_ = 0.002, *p* =0.99; ***p < 0.001 vs saline in *post hoc* test). All bars represent mean ± standard error of the mean (SEM).

The short- and long-term behavioral effects of S-ketamine in rats were examined in three tests. The NSF test, which is sensitive to long-term but not to short-term monoaminergic antidepressant treatment (Bodnoff et al., 1988), was used to assess the short-term effects of ketamine at three different doses. The injection of 10 mg/kg ketamine 1 h prior to testing significantly reduced the latency to feed in the novel environment by 47% (one-way ANOVA, *F*_(3,14)_ = 6.61, *p* = 0.005; *Holm–Sidak test, 10 mg/ml vs saline, *p* = 0.004; Fig. 1*A*), as previously observed with the racemic mixture (Li et al., 2010; Carrier and Kabbaj, 2013). Lower doses of 2.5 and 5 mg/kg had no effect, in contrast to what has been reported using the 5 mg/kg racemic mixture (Carrier and Kabbaj, 2013).

The forced swim test (FST) is used classically to detect antidepressant activity in rodents following short-term treatment (Porsolt et al., 2001). Repeated FST, which can detect behavioral changes following long-term treatment with low doses of classic antidepressants (Cryan et al., 2005), was used to assess the sustained antidepressant effect of ketamine (Fig. 1*B*). Short-term administration of ketamine (10 mg/kg, i.p.; 24 h, 4 h, and 30 min prior to testing) significantly decreased immobility time by 30% in the first test and by 20% 21 d later (main effect of treatment: *F*_(2,9)_ = 31.65, *p* = 0.0001; main effect of time: *F*_(1,9)_ = 31.47, *p* = 0.0003; treatment × time interaction: *F*_(2,9)_ = 0.002, *p* =0.99; ketamine vs saline: *p* = 0.0007, first session; *p* = 0.0006, second session). Treatment with the typical SSRI fluoxetine, at a dose showing long-term but not short-term effects in previous studies (Porsolt et al., 2001; Cryan et al., 2005), produced no effect in either session. These results indicate that low-dose ketamine, unlike fluoxetine, produces antidepressant-like effects that begin within 1 d and last at least 3 weeks, extending the time course previously observed in mice (Maeng et al., 2008).

### Ketamine rapidly accelerates functional maturation of new neurons in the dentate gyrus

**Figure 2.**
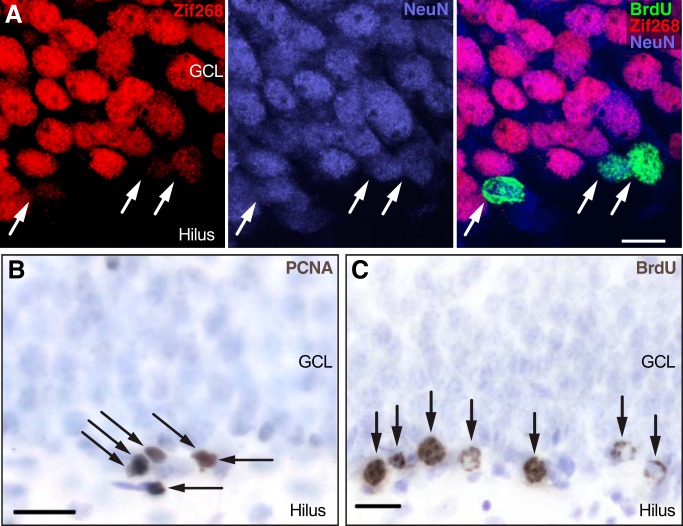
Examples of immunohistochemical markers. ***A***, After kainate injection, all mature granule neurons and some BrdU-labeled 16-d-old NeuN^+^ neurons expressed zif268, indicating synaptic activation. GCL, granule cell layer ***B***, Dividing cells (arrows) were identified using PCNA immunohistochemistry. ***C***, Cells surviving 2-3 weeks (arrows) were identified with BrdU immunohistochemistry (gray-brown); immunonegative cells were stained with blue-purple counterstain.

**Figure 3.**
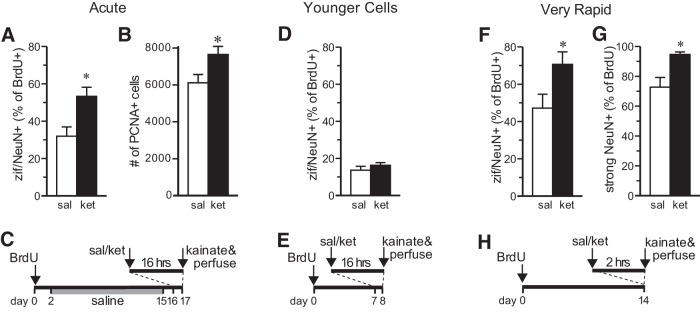
Rapid effects of ketamine on granule cell maturation and proliferation. ***A***, S-ketamine (ket) increased the proportion of 16-d-old BrdU^+^ cells colabeled with NeuN and zif268 (zif) 16 h later, relative to saline-treated controls (sal) (*t test, *t*_(4)_ = 3.065, *p* =0.0375). All bars represent mean ± SEM. ***B***, S-ketamine increased the number of PCNA^+^ (dividing) cells in the subgranular zone 16 h later (*t test, *t*_(10)_ = 2.42, *p* = 0.0359). ***C***, Animal treatment time course for short-term effects; ketamine injection was 10 mg/kg, i.p., in each experiment. ***D***, The maturation effect was not seen in 7-d-old cells (*t* test, *t*_(9)_ = 0.98, *p* = 0.35). ***E***, Animal treatment time course for short-term effects in young cells. ***F***, ***G***, Increased zif/NeuN coexpression and strong NeuN expression were seen in 14-d-old cells within 2 h of ketamine treatment (zif: *t test, *t*_(9)_ = 2.33, *p* = 0.0450; strong NeuN: *t test, *t*_(9)_ = 3.55, *p* = 0.0062). ***H***, Animal treatment time course for very rapid effects on maturation.

Kainate induced strong expression of zif268 throughout the granule cell layer in both groups (Fig. 2*A*). In control rats (Fig. 3*A*), 32% of the 16-d-old neurons expressed zif268 in response to kainate activation, consistent with expectations for rat granule neurons at this time point (Snyder et al., 2009b). In ketamine-treated rats, the proportion of BrdU^+^/NeuN^+^ cells labeled with zif268 was 67% higher (*t* test, *t*_(4)_ = 3.065, *p* = 0.0375; Fig. 3*A*), suggesting a rapid increase in the synaptic integration of 2-week-old granule cells. The 50 min half-life of ketamine in the rat brain (Mathew et al., 2012) is short relative to the 16 h post-ketamine administration delay in this experiment, arguing against any direct interaction between ketamine and kainate.

Increased granule cell precursor proliferation is a common feature of antidepressant treatments (Duman et al., 2001) and could play a role in long-term behavioral effects of ketamine. Mitotic cells were assayed 16 h after ketamine injection using the endogenous marker PCNA (Fig. 2*B*). Short-term administration of ketamine significantly increased the number of PCNA^+^ cells located in the subgranular zone by 25% (*t* test, *t*_(10)_ = 2.42, *p* = 0.0359; Fig. 3*B*), which is consistent with the increased cell proliferation produced by other NMDA receptor antagonists (Cameron et al., 1995; Nacher et al., 2001; Nacher and McEwen, 2006).

To test the maturation effects in younger cells, the experiment was repeated by administering BrdU only 7 d prior to ketamine injection (Fig. 3*E*). There was no effect of ketamine on zif268 expression in these 7-d-old cells (*t* test, *t*_(9)_ = 0.98, *p* = 0.35; Fig. 3*D*), supporting the specificity of kainate-induced IEG expression as a measure of synaptic integration and suggesting that new maturation can only be rapidly accelerated after they have reached a certain level of maturity.

Behavioral effects of ketamine have been observed within 2 h of ketamine administration (Fig. 1; Garcia et al., 2008; Engin et al., 2009; Li et al., 2010), so functional maturation of 14-d-old granule cells was assessed at this very short time point. After 2 h, ketamine increased the proportion of 14-d-old cells expressing zif268 by 50% compared with saline (*t* test, *t*_(9)_ = 2.33, *p* = 0.0450; Fig. 3*F*). Ketamine also significantly increased the proportion of BrdU^+^ cells strongly immunoreactive for NeuN (*t* test, *t*_(9)_ = 3.55, *p* = 0.0062; Fig. 3*G*), another measure of neuronal maturity (Snyder et al., 2009b). Together, these data demonstrate that a low dose of ketamine very rapidly induces the maturation of young granule cells.

### Long-term daily ketamine treatment does not enhance single-injection effects

**Figure 4.**
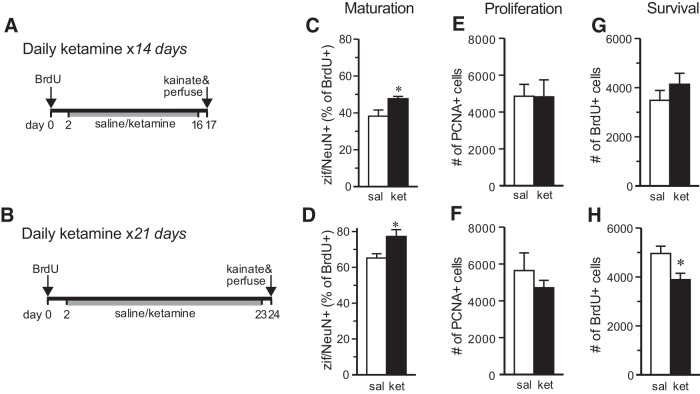
Effects of long-term ketamine treatment. ***A***, ***B***, Animal treatment time courses; all ketamine injections were 10 mg/kg, i.p. ***C***, ***D***, Long-term daily ketamine treatment for 14 d (***C***) or 21 d (***D***) increased the proportion of zif/NeuN^+^ BrdU^+^ granule cells (*14 d: *t* test, *t*_(9)_ = 3.20, *p* = 0.0108; *21 d: *t* test, *t*_(11)_ = 2.773, *p* = 0.0181). ***E***, ***F***, S-ketamine had no effect on cell proliferation when administered daily for 14 or 21 d (14 d: *t*_(10)_ = 0.035, *p* = 0.973; 21 d: *t*_(9)_ = 0.955, *p* = 0.365). ***G***, ***H***, BrdU^+^ cell survival was unaffected by 14 d of daily treatment with S-ketamine (*t* test, *t*_(10)_ = 1.03, *p* = 0.33) but was decreased after 21 d (*t test, *t*_(12)_ = 2.71, *p* = 0.0191). All bars represent the mean ± SEM (*n* = 6-7 per group).

Several studies have examined the behavioral effects of long-term daily ketamine treatment (Browne and Lucki, 2013). To determine the effects of long-term treatment with ketamine on neurogenesis, animals received ketamine daily (10 mg/kg, i.p.) for 15 or 22 d, with kainate injection and perfusion 16 h after the last ketamine injection (Fig. 4*A*,*B*). Long-term ketamine significantly increased the proportion of BrdU-NeuN-zif268^+^ cells compared with the saline-treated group at both time points (14 d: *t* test, *t*_(9)_ = 3.20, *p* = 0.0108; 21 d: *t* test, *t*_(11)_ = 2.773, *p* = 0.0181; Fig. 4*C*,*D*). These increases, however, were approximately half as large as those seen with short-term treatment. PCNA cell counting showed no effect of long-term ketamine treatment after 14 or 21 d (14 d: *t*_(10)_ = 0.035, *p* = 0.973; 21 d: *t*_(9)_ = 0.955, *p* = 0.365; Fig. 4*E*,*F*).

The effects of ketamine on the survival of new granule cells was examined after long-term treatment by counting BrdU-labeled cells. To isolate the effects on survival from possible proliferation effects (Dayer et al., 2003), rats were given BrdU 2 d before ketamine treatment began. Long-term treatment with ketamine for 14 d had no effect on the number of surviving BrdU^+^ cells located in the granule cell layer (*t* test, *t*_(10)_ = 1.03, *p* = 0.33; Fig. 4*G*), which is consistent with the lack of effect of the NMDA receptor antagonist 3-(2-carboxypiperazin-4-yl)propyl-1-phosphonic acid (CPP) on the survival of young granule cells observed previously in mice (Tashiro et al., 2006). Interestingly, 21 d of ketamine treatment decreased new granule cell survival (*t* test, *t*_(12)_ = 2.71, *p* = 0.0191; Fig. 4*H*), producing the only negative effect of ketamine on neurogenesis observed in the study.

### Prolonged effects in a depression model

**Figure 5.**
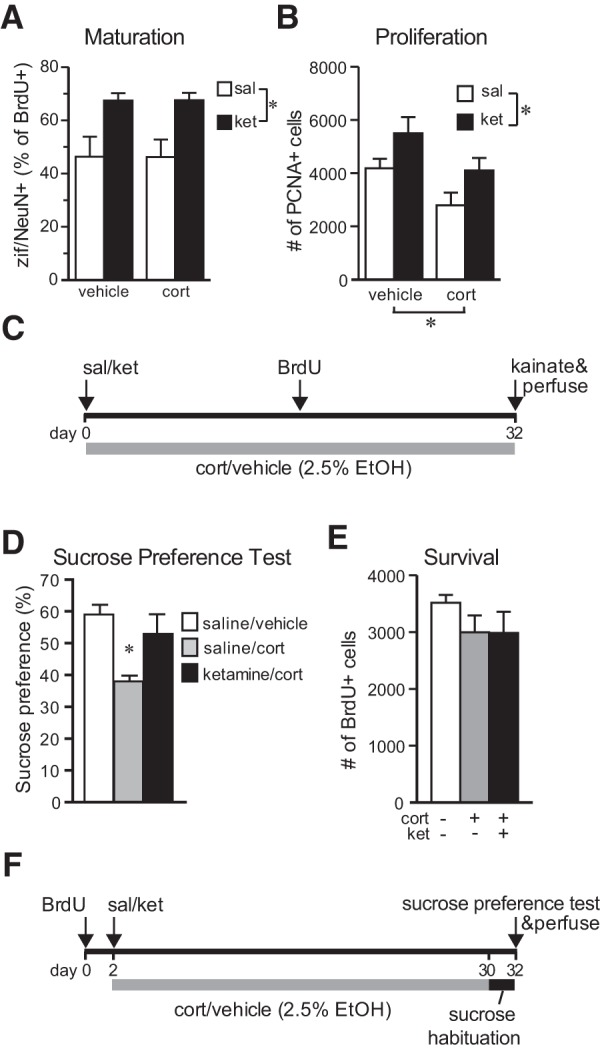
Sustained effects of S-ketamine in a depression model. ***A***, Ketamine given 32 d earlier increased zif/NeuN expression in 16-d-old cells regardless of long-term corticosterone exposure (main effect of CORT: *F*_(1,17)_ = 0.00, *p* = 0.996; *main effect of ketamine: *F*_(1,17)_ = 14.99, *p* = 0.0017; CORT × ketamine interaction: *F*_(1,17)_ = 0.0005, *p* = 0.9821 by two-way ANOVA). ***B***, S-ketamine increased the number of PCNA^+^ cells 32 d later and prevented the inhibition of proliferation by long-term corticosterone treatment (*main effect of ketamine: *F*_(1, 23)_ = 7.44, *p* = 0.013; *main effect of CORT: *F*_(1,23)_ = 8.35, *p* = 0.009; CORT × ketamine interaction: *F*_(1,23)_ = 0.00, *p* = 0.988 by two-way ANOVA). ***C***, Animal treatment time course for maturation and proliferation effects. ***D***, A single S-ketamine injection prior to long-term CORT treatment prevented a decrease in sucrose preference (one-way ANOVA, *F*_(2,15)_ = 6.98, *p* = 0.0072; *p < 0.05 vs saline in *post hoc* test). Values represent the mean ± SEM (*n* = 4-6 per group). ***E***, Neither long-term exposure to CORT nor short-term ketamine exposure prior to CORT significantly altered new cell survival (*F*_(2,15)_ = 1.12, *p* = 0.35 by one-way ANOVA). Values represent the mean ± SEM (*n* = 6-7 per group. ***F***, Animal treatment time course for sucrose preference and survival effects.

To assess the duration of the effects of a single injection of ketamine, maturation and proliferation were examined 32 d after a single injection of ketamine (10 mg/kg, i.p) or saline. Ketamine effects were tested in control conditions and in a depressive-like state (Sterner and Kalynchuk, 2010) induced by long-term CORT treatment (Fig. 5*C*). CORT had no effect on zif268 expression, suggesting that maturation is unaffected by excess glucocorticoids (Fig. 5*A*). Ketamine, however, significantly increased the proportion of 16-d-old cells expressing zif268 by 45% in both CORT-treated and untreated rats (main effect of CORT: *F*_(1,17)_ = 0.00, *p* = 0.996; *main effect of ketamine: *F*_(1,17)_ = 14.99, *p* = 0.0017; CORT × ketamine interaction: *F*_(1,17)_ = 0.0005, *p* = 0.982 by two-way ANOVA), indicating that ketamine continues to accelerate neuronal maturation for several weeks, even in neurons born long after short-term ketamine treatment.

A single ketamine injection increased cell proliferation 32 d later (main effect of ketamine: *F*_(1, 23)_ = 7.44, *p* = 0.013; Fig. 5*B*,*C*). This finding extends a previous report that the NMDA receptor antagonist CGP43487 increases cell proliferation for at least 7 d (Nacher et al., 2001), but contrasts with a recent study showing no effect of ketamine on cell proliferation or DCX^+^ cells 29 d after ketamine injection (Brachman et al., 2015), suggesting either a species difference or decreased efficacy of higher ketamine doses. Long-term exposure to corticosterone decreased the number of PCNA^+^ cells by 33% (main effect of CORT: *F*_(1, 23)_ = 8.35, *p* = 0.009; Fig. 5*B*), as expected based on previous studies (Wong and Herbert, 2005; David et al., 2009). CORT and ketamine had independent, additive effects that, when combined, resulted in a proliferation rate very close to the control level (CORT × ketamine interaction: *F*_(1, 23)_ = 0.00, *p* = 0.988 by two-way ANOVA; Fig. 5*B*). A previous study found interactive effects, suggesting that NMDA receptor activation acts downstream of corticosterone at short-term time points (Cameron et al., 1998). The results observed here suggest that the sustained effects of ketamine, after the drug itself is out of the system, may not directly involve NMDA receptors.

To determine the effects of ketamine on anhedonia and survival, the experiment was repeated, administering BrdU prior to treatment and testing for sucrose preference (Fig. 5*F*), a model of anhedonia (David et al., 2009; Sterner and Kalynchuk, 2010). Rats treated with corticosterone for 28 d showed a 30% decrease in preference for sucrose compared with saline/vehicle-treated animals (Fig. 5*D*; for review, see Gourley et al., 2009; Sterner and Kalynchuk, 2010). This effect was reversed by ketamine (10 mg/kg, i.p.) given 32 d before testing (one-way ANOVA, *F*_(2,15)_ = 6.98, *p* = 0.0072; *p* = 0.0405 vs saline/CORT in *post hoc* test; Fig. 5*D*), a change that is unlikely to have been produced by nonspecific changes in thirst or hunger, because body weights and the total volume consumed during the 1 h test (∼30 ml) showed no significant group differences (*p* = 0.61 and *p* = 0.22, respectively). This experiment demonstrates sustained antidepressant activity of a single dose of ketamine in a model of depression that requires long-term treatment for efficacy of classic antidepressants (Sterner and Kalynchuk, 2010). These results are consistent with recently observed ketamine effects on NSF in a similar paradigm (Brachman et al., 2015).

No statistically significant effects of either CORT or ketamine on BrdU^+^ cell survival were observed (*F*_(2,15)_ = 1.12, *p* = 0.35 by one-way ANOVA; Fig. 5*E*). The data suggest a possible inhibitory effect of corticosterone on survival but provide no hint of any effect of ketamine.

### New neurons are not required for behavioral effects of ketamine on neophagia

**Figure 6.**
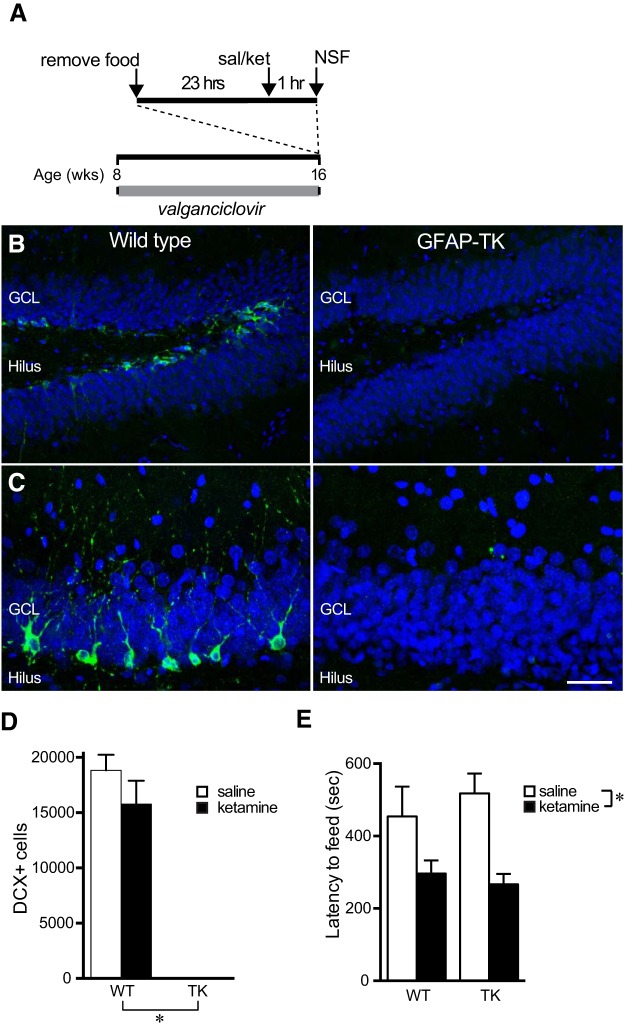
Neurogenesis is not required for the S-ketamine effect on novelty-suppressed feeding. ***A***, Animal treatment time course showing valganciclovir to inhibit neurogenesis, the injection of saline (sal) or ketamine (ket; 10 mg/kg), and NSF testing. ***B***, Photographs show DCX-expressing young granule neurons (green) in the dentate gyrus of valganciclovir (VGCV)-treated wild-type rats (WT), but not in GFAP-TK (TK) rats. Blue counterstain shows cell nuclei. ***C***, Higher magnification of granule cell layer showing DCX staining. ***D***, Quantification shows near-complete absence of DCX^+^ new neurons in GFAP-TK rats and no effect of short-term S-ketamine treatment on DCX^+^ cell number. (*main effect of genotype: *F*_(1,20)_ = 183.6, *p* < 0.0001; main effect of ketamine: *F*_(1,20)_ = 1.471, *p* = 0.2392; genotype × ketamine interaction: *F*_(1,20)_ = 1.418, *p* = 0.2477, all by two-way ANOVA) ***E***, In the NSF test, the latency to eat in a novel arena was decreased by S-ketamine in both wild-type and GFAP-TK rats (*main effect of genotype: *F*_(1,20)_ = 0.2827, *p* = 0.6008; main effect of ketamine: *F*_(1,20)_ = 13.24, *p* = 0.0016; genotype × ketamine interaction: *F*_(1,20)_ = 0.3756, *p* = 0.5469, all by two-way ANOVA). All bars represent mean ± SEM.

To investigate whether rapid changes in the maturation of young neurons are causally related to rapid antidepressant-like effects in rats, we tested the behavioral effects of S-ketamine on NSF in rats lacking adult neurogenesis (Fig. 6*A*). This test was chosen because we and others have seen an antidepressant effect of ketamine in this test and because neurogenesis-dependent effects of fluoxetine have been found in this test (Santarelli et al., 2003; David et al., 2009). After 8 weeks of valganciclovir treatment, which virtually eliminated new neurons in the dentate gyrus of GFAP-TK rats (Fig. 6*B*), NSF behavior was tested in GFAP-TK rats and wild-type littermate controls. Injection of 10 mg/kg ketamine 1 h prior to testing significantly reduced the latency to feed in the novel environment (main effect of treatment in two-way ANOVA: *F*_(1,22)_ = 13.47, *p* = 0.002; Fig. 6*C*), as seen in our initial experiment (Fig. 1*A*). There was no main effect of genotype or treatment × genotype interaction (main effect of genotype: *F*_(1,22)_ = 0.09, *p* = 0.767; treatment × genotype interaction: *F*_(1,22)_ = 0.71, *p* = 0.411 by two-way ANOVA), indicating that new neurons are not required for the behavioral effects of S-ketamine in this test. Home cage consumption was not measured in this experiment, but previous studies in rats and mice have found no effect of short-term ketamine treatment on this measure (Autry et al., 2011; Li et al., 2011; Iijima et al., 2012; Gideons et al., 2014; Nosyreva et al., 2014). Neurogenesis-dependent effects on sucrose preference and forced swim behavior at baseline, in the absence of ketamine, prevented testing of the requirement for neurogenesis in the antidepressant effects of ketamine in these tests (Snyder et al., 2011).

## Discussion

The present findings demonstrate that S-ketamine has both rapid and sustained effects on adult neurogenesis in the dentate gyrus. A single injection of ketamine increased the functional maturation of young neurons within hours and continued to accelerate maturation for at least 4 weeks. An increase in cell proliferation was also observed shortly after ketamine treatment and was sustained for at least 4 weeks. Long-term daily treatment with ketamine had more limited effects and had a small but significant negative effect on new neuron survival. The rapid and prolonged cellular effects matched the time course of behavioral effects in the NSF test, the FST, and the sucrose preference test. However, direct testing of the relationship between neurogenesis and behavioral effects on neophagia showed that new neurons were not required for a ketamine-induced decrease in this depressive-like behavior.

Accelerated maturation of adult-born neurons has previously been observed, but only after treatments lasting ≥1 weeks. Treatment of mice with fluoxetine for 28 d increases newborn neuron dendritic length, while 5 d of treatment does not (Wang et al., 2008). Agomelatine, a melatonergic receptor agonist and 5-HT_2C_ receptor antagonist, accelerates NeuN expression in granule cells after 8 d (Soumier et al., 2009). A nonpharmacologic treatment, exercise, increases the proportion of mature young neurons after 21 d, but not after 14 d (Snyder et al., 2009a). The current study shows that similar changes in maturation can be induced by ketamine within only 2 h. Many studies have demonstrated the formation of new synapses in the adult brain within days (Woolley and McEwen, 1992; Holtmaat and Svoboda, 2009), but the effects of ketamine on circuit formation are surprisingly fast, even by this standard. However, *in vitro* studies have demonstrated that dendritic spines on neocortical pyramidal neurons can form *de novo* in response to glutamate within minutes (Kwon and Sabatini, 2011; Okabe, 2013).

Ketamine acts at NMDA receptors, which are found throughout the brain. Our experiments could not determine whether the key NMDA receptors are those on the new neurons themselves or whether the effect is indirect. Studies using specific antagonists suggest that the effects of ketamine on synapse formation and depressive-like behavior are mediated through the NMDA receptors containing the NR2B subunit (Maeng et al., 2008; Li et al., 2010). NR2B-containing NMDA receptors, generally thought of as a developmental form of the NMDA receptor, are expressed on young granule cells and are required for a form of plasticity produced exclusively by young neurons (Snyder et al., 2001). Therefore, it is reasonable to suspect that the effects of ketamine on maturing granule cells occur directly through NR2B-containing NMDA receptors on these young neurons. Deletion of the NR1 subunit of NMDA receptors from young neurons decreases their survival in a cell-specific manner (Tashiro et al., 2006), supporting a direct role for NMDA receptors on the young neurons. The decrease in new neuron survival seen following 21 d of treatment with ketamine is consistent with the genetic ablation effects, though the relatively small effects seen with ketamine treatment suggest that survival effects of transient blockade are small and may be offset by increased cell proliferation.

Because ketamine acts as an antagonist, blocking NMDA receptors, the current findings suggest that endogenous activation of NMDA receptors normally slows the incorporation of new neurons into functional circuits. Neuronal activation is generally regarded as being an important positive modulator of neuronal maturation, so the enhancement of maturation by NMDA receptor blockade is somewhat counterintuitive. However, genetic ablation of NMDA receptors in developing CA1 pyramidal cells increases the number of functional synapses detected by slice physiology (Adesnik et al., 2008), which is counter to expectations but is consistent with our results. Developing neurons, including adult-born granule neurons (Chancey et al., 2013), have many silent synapses containing NMDA receptors, but no AMPA receptors. According to the model developed by Adesnik et al. (2008), low-level stimulation of these NMDA receptors inhibits AMPA receptor trafficking to the postsynaptic density. Strong activation of NMDA receptors normally overcomes this inhibition at some point during development, but the deletion of NMDA receptors, or perhaps in our case pharmacological blockade of the NMDA receptors, disinhibits AMPA receptor trafficking and results in greater numbers of AMPA receptor-containing functional synapses. This AMPA receptor trafficking and the stabilization of synapses occurs within minutes (Groc et al., 2006). This model is supported by findings that AMPA receptor activation is required for the antidepressant effects of ketamine (Maeng et al., 2008; Li et al., 2010; Autry et al., 2011; Koike et al., 2011). BDNF, which is upregulated by ketamine and has been suggested as a mediator of its antidepressant effects (Garcia et al., 2008; Duman, 2014), also increases CA1 pyramidal cell dendritic spine size, an indicator of synapse maturity, within 10 min (Hale et al., 2011).

The finding that ketamine decreases feeding latency in the novelty-suppressed feeding task in adult rats lacking adult neurogenesis indicates that new neurons are not required for the antidepressant-like effects of ketamine, at least in this task. No rodent model of depressive-like behavior is clearly predictive of efficacy or the mechanism of action in humans (Nestler and Hyman, 2010; Fernando and Robbins, 2011; Hyman, 2012), so it is possible that neurogenesis could play a role in the antidepressant activity of ketamine in humans. However, NSF may be the best available test for assessing ketamine effects on depressive-like behavior in rodents for several reasons. First, behavioral changes in NSF faithfully model the time course of antidepressant effects in humans, requiring long-term treatment with SSRIs but only short-term treatment with ketamine. In addition, SSRI effects on NSF behavior, in contrast to those of ketamine, do require new neurons in mice (Santarelli et al., 2003), demonstrating a clear distinction from the ketamine effects on this test. This difference suggests that ketamine and SSRIs act through a different mechanism, not just at the level of receptors and neurotransmitter systems, but also at the level of neuronal populations. These findings further suggest that changes in adult neurogenesis are not the final common pathway for all antidepressant effects on rodent behavior and may not be important for the therapeutic effects of ketamine in humans. The effects of ketamine on behavior can be blocked with drug infusions into the prefrontal cortex (Li et al., 2010), suggesting that this brain region does play a role in antidepressant effects. Changes in AMPA receptors, signaling molecules, and dendritic spines have been observed in the prefrontal cortex 24 h after ketamine treatment (Browne and Lucki, 2013; Duman, 2014) but have not been described at earlier time points. Nonetheless, very rapid effects on synapse maturation, like those observed in the current study, may also occur in presumably mature neurons in the prefrontal cortex and could provide a mechanism for the behavioral effects. It may be difficult to capture these very rapid changes in mature neurons, where only a subset of spines is normally silent or otherwise immature and able to be modified.

Although ketamine does not appear to act through new neurons to produce its antidepressant effects in the NSF test, albeit with the caveats discussed above, its proneurogenic effects should be considered a side effect of this therapeutic treatment. The sustained effects, including increased cell proliferation and maturation for at least 4 weeks after a single treatment, may be the most important effects to consider in this context. Whether this increase in new neurons and the acceleration of their maturation would be expected to have positive or negative effects on mental health is not yet clear. Nor is it apparent whether the effects on adult neurogenesis are important for the sustained behavioral effects of ketamine, which was not directly tested in this study. However, the rapid effects of ketamine on new neurons may be valuable both for understanding the normal maturation of adult-born neurons and for studying the synaptic effects of ketamine that may occur more globally including in neurons responsible for its antidepressant effects.

## Synthesis

The decision was a result of the Reviewing Editor Liisa Galea and the peer reviewers coming together and discussing their recommendations until a consensus was reached. A fact-based synthesis statement explaining their decision and outlining what is needed to prepare a revision is listed below. The following reviewers agreed to reveal their identity: Jonathan Epp, Cheryl Sisk.

This manuscript examines the mechanisms underlying the antidepressant effects of ketamine. The mechanism explored related to adult neurogenesis. The authors confirm that ketamine does have an impact on the proliferation and maturation of new neurons. However, blocking neurogenesis does not interfere with the ability of ketamine to produce antidepressant like effects in a novelty suppressed feeding task.

In general the reviewers agreed that the experiments were well performed and the manuscript was well written. There are a few changes and considerations that are requested.

1)The authors should quantify doublecortin expression in their TK experiment as well as provide lower magnification images of the dentate. The actual level of neurogenesis depletion is not clear from the images provided.

2)Statistics need to be given in the results section - e.g. along with the statements that there is a reduction of xx% please also state a p value and/or effect size.

3)The conclusions need to be tempered (also in title, abstract and significance statement) as the conclusion that neurogenesis is not required for the antidepressant efficacy of ketamine was done only in the NSF task in the GFAP-TK rats. The authors began by documenting that ketamine has effects on several depressive like behaviors. Thus it theoretically would be more convincing to see that ketamine doesn't require neurogenesis for any of these other behaviors. Indeed, NSF is classically considered a test of anxiety-like behavior not a test of depressive-like behavior (although antidepressants do reduce latency to feed - as do anxiolytics). As such many statements need to be weakened due to the examination of only one behavior.

4)The authors need to acknowledge that while the authors show that ketamine has both fast acting and persistent behavioural effects the experiments only the attempt to block the immediate effects by eliminating neurogenesis. This leaves the possibility that the long-lasting effects of ketamine require neurogenesis. This should be acknowledged.

5)Line 441: insert "of" at end of this line.

6)It is important to examine food consumption data for after NSF (as this is needed to indicate that the treatment had no effect on satiety that may interfere with interpretation of results - usually this is done by recording food consumption in the home cage for a few minutes after the test. If this was done please provide the data.

7)Line 123: Specify route of administration of S-ketamine.

8)Line 216 (or elsewhere): Provide a brief explanation of what zif268 is and why it is an appropriate marker to use in this context.
